# Coverage–Dependent Structural Evolution of CoBr_2_ at the Au(111) Interface

**DOI:** 10.1002/advs.202508262

**Published:** 2025-11-14

**Authors:** Samuel Kerschbaumer, Martin Ondráček, Sebastien E. Hadjadj, Oleksandr Stetsovych, Andrés Pinar Solé, Adriana Elizabet Candia, Paula Angulo‐Portugal, Andrea Aguirre‐Baños, Martina Corso, David Serrate, Jorge Lobo‐Checa, Pavel Jelínek, Maxim Ilyn, Pablo M. Piaggi, Celia Rogero

**Affiliations:** ^1^ Centro de Física de Materiales (CSIC/UPV‐EHU) 20018 Donostia‐San Sebastián Spain; ^2^ FZU ‐ Institute of Physics of the Czech Academy of Sciences Cukrovarnická 10 Prague 6 CZ 16200 Czech Republic; ^3^ Laboratorio de Microscopias Avanzadas (LMA) Universidad de Zaragoza Zaragoza E‐50018 Spain; ^4^ Instituto de Nanociencia y Materiales de Aragón (INMA) CSIC‐Universidad de Zaragoza 50009 Zaragoza Spain; ^5^ Departamento de Física de la Materia Condensada Universidad de Zaragoza E‐50009 Zaragoza Spain; ^6^ CIC nanoGUNE‐BRTA Tolosa Hiribidea, 76 Donostia‐San Sebastián 20018 Spain; ^7^ Ikerbasque Basque Foundation for Science 48013 Bilbao Spain

**Keywords:** 2D magnetic materials, CoBr_2_ single layer growth, transition metal dihalides, van der Waals semiconductors

## Abstract

Unraveling the growth mechanism of van der Waals materials is crucial for their device implementation, as this improves the overall film quality, allowing precise control of their electronic and magnetic properties in nanoscale applications. The initial structure formed on the substrate during growth is often assumed to be bulk‐like, thereby neglecting the role of the surface in the assembly. Here, the coverage–dependent growth of CoBr_2_ on Au(111) from a stoichiometric molecular powder is studied using a combination of experimental techniques, machine–learning‐driven molecular dynamics simulations and density functional theory calculations. It is found that CoBr_2_ molecules initially form a molecular precursor phase characterized by three‐molecule clusters arranged in a surface–stabilized structure with long‐range order and a periodic coincidence with Au(111). As the surface coverage is increased, this phase subsequently undergoes a transition to form the equilibrium van der Waals crystal layered structure observed for the bulk material. These findings challenge conventional views of direct van der Waals layer formation and provide new insight into the role of the substrate during the growth process.

## Graphical abstract

CoBr_2_ grown on Au(111) initially forms a surface‐stabilized molecular precursor phase is composed of ordered three‐molecule clusters that lie flat on the surface and have a periodic coincidence with the substrate. As coverage increases, the precursor phase transforms into the bulk‐like van der Waals 1T trilayer, with subsequent layers adopting the same bulk‐like structure, challenging conventional views of direct van der Waals layer formation.

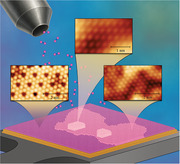

## 1 Introduction

2D materials and their potential applications in advanced technologies have made them a focal point in materials science research. A particular class of 2D materials that has attracted significant attention are magnetic 2D materials, especially transition metal dichalcogenides (TMDs)^[^ ^1^, ^2^, ^3^ ^]^ and transition metal dihalides (TMDHs) and trihalides (TMTHs).^[^ ^4^, ^5^, ^6^ ^]^ Many of these materials crystallize in van der Waals (vdW) layered structures, making them easily cleavable,^[^ ^7^, ^8^ ^]^ while their partially filled d‐orbitals can lead to magnetic order, making them promising candidates for spintronics and other applications where magnetic order is required.^[^ ^9^, ^10^, ^11^ ^]^ TMDHs are also highly sought after due to their gate tunability in field–effect transistors and their layer by layer growth from a molecular stoichiometric compound at relatively low evaporation temperatures (≈400 °C), often essential for developing custom functional heterostructures and devices.^[^ ^12^, ^13^ ^]^

**Figure 1:**
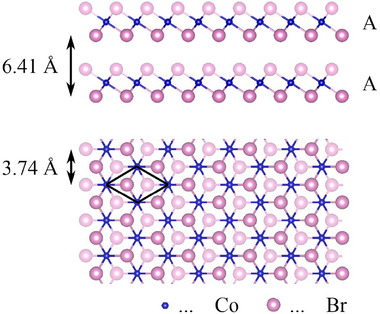
Schematic representation of bulk CoBr_2_, shown in side and top views. The layer height and unit cell dimensions are indicated, illustrating that each monolayer is composed of three atomic planes arranged in an octahedral 1T configuration. In the bulk material, CoBr_2_ adopts an AA stacking sequence, where successive monolayers are aligned directly on top of each other.

In recent years, theoretical calculations^[^ ^4^, ^7^, ^14^, ^15^, ^16^, ^17^, ^18^, ^19^ ^]^ and experimental reports^[^ ^20^, ^21^, ^22^ ^]^ for various TMDHs have shown magnetic order down to the single layer limit. For synthesizing high‐quality films, it is crucial to understand the growth mechanism of these materials and the influence of substrate interactions on the growth process. In bulk, these materials crystallize in the 1T structure: each monolayer consists of three atomic planes stacked in an X–M–X sequence (for example **Figure** **1** shows the structure for CoBr_2_: Br–Co–Br), with the metal atoms forming a hexagonal planar lattice, each octahedrally coordinated by six halogen atoms. For consistency to pre‐existing literature we refer to it as monolayer (ML) CoBr_2_. However, within this family, FeBr_2_ has recently been reported to form an initial surface‐stabilized phase on Au(111) that does not correspond to the bulk 1T crystal structure.^[^ ^21^, ^23^ ^]^ This indicates that early stages of growth in TMDHs may involve other configurations distinct from the bulk‐like phase. Determining whether the growth follows molecular assembly, substrate/adatom mediation or direct monolayer formation allows for precise growth control, minimizing defects and ensuring higher‐quality films with improved reproducibility and enhanced performance. This is essential for fine‐tuning synthesis parameters to engineer material characteristics for specific applications, such as in spintronics or quantum devices, and it is particularly important for scaling up fabrication processes, where batch–to–batch consistency is critical.

In this work, we present a two‐step growth model for CoBr_2_ on Au(111) based on an experimental and theoretical multi‐technique approach. By analyzing structural features across different coverages we show that small inorganic molecules initially form a molecular precursor phase (PP). This phase is characterized by an ordered and periodic structure, which extends over large surface areas before reorganizing into the bulk‐like ML structure at higher coverages. The PP phase displays a surface reconstruction analogous to that reported for FeBr_2_,^[^ ^21^, ^23^ ^]^ reinforcing the idea that such reconstructed phases could represent a more general stabilization mechanism in this material class. Our AFM Z–spectroscopy measurements together with machine–learning–driven molecular dynamics simulations demonstrate that this PP originates from a surface–stabilized molecular arrangement of CoBr_2_ units, which reorganizes into the bulk–like 1T monolayer at higher coverages. These results point to a new growth mechanism that differs from existing interpretations.^[^ ^23^ ^]^ The growth behavior of CoBr_2_ serves as a model system for understanding how precursor phases might form in other TMDH and vdW materials, guiding the development of new heterostructures with tailored interfacial properties.

## 2 Results and Discussion

**Figure 2:**
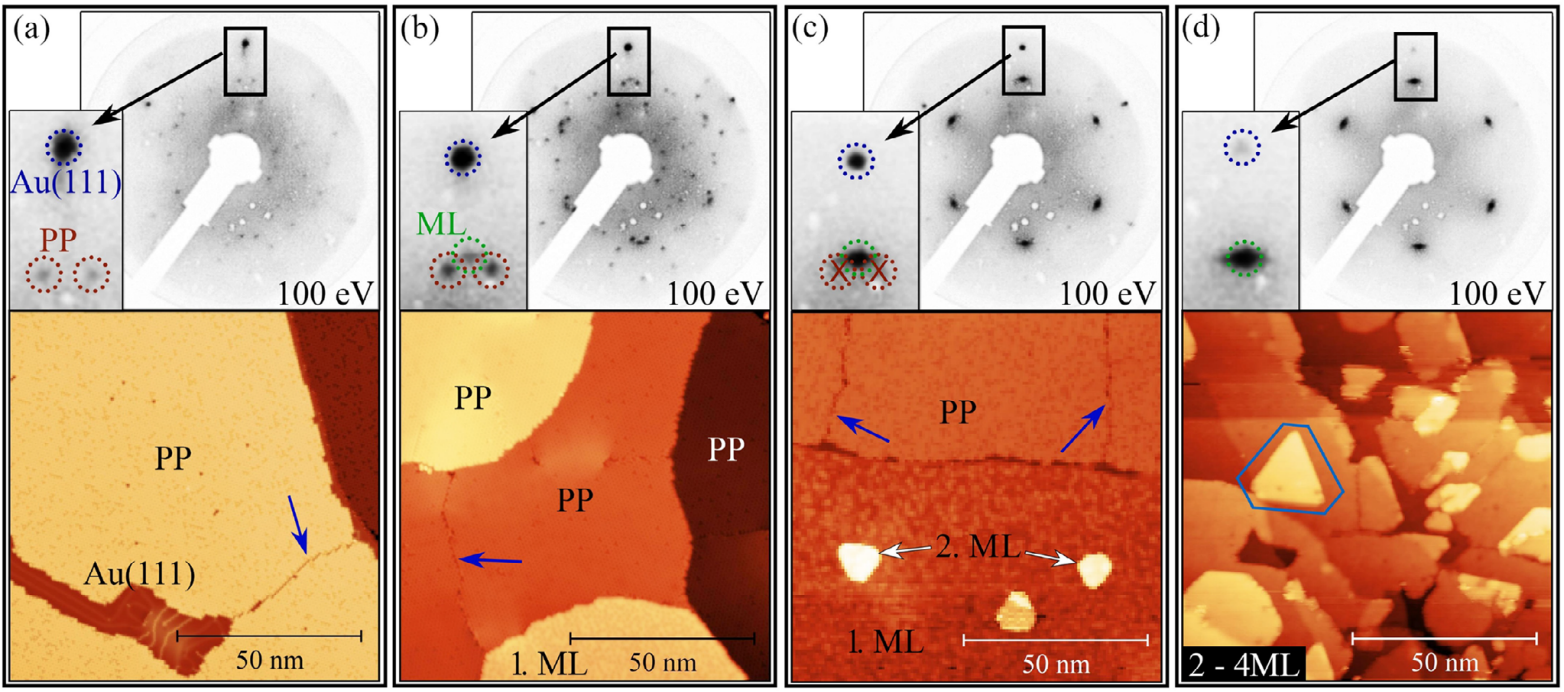
STM images and LEED patterns of CoBr_2_ growth on Au(111) with increasing coverage: a) sub–monolayer (only PP), b) PP and first monolayer (1. ML), c) coexistence of PP, 1. ML and 2. ML island nucleation on top of the 1. ML and d) multilayer (2‐4 ML) islands. The colored circles in the LEED zoom mark Au(111) (blue), the two domains of the PP (brown, ±5° relative to the Au(111)) and the 1. ML and 2. ML (green, aligned to Au(111)) spots; blue arrows in STM indicate domain boundaries. From the 2. ML onward, CoBr_2_ forms large, partially transparent islands with hexagonal symmetry (blue hexagon) that seamlessly overgrow step edges. STM Imaging conditions: (a) *U* = 1.0 mV, *I*_*t*_ = 50 pA; (b) *U* = 1.2 mV, *I*_*t*_ = 20 pA; (c) *U* = 1.5 mV, *I*_*t*_ = 30 pA; (d) *U* = 1.5 mV, *I*_*t*_ = 10 pA.

**Figure 3:**
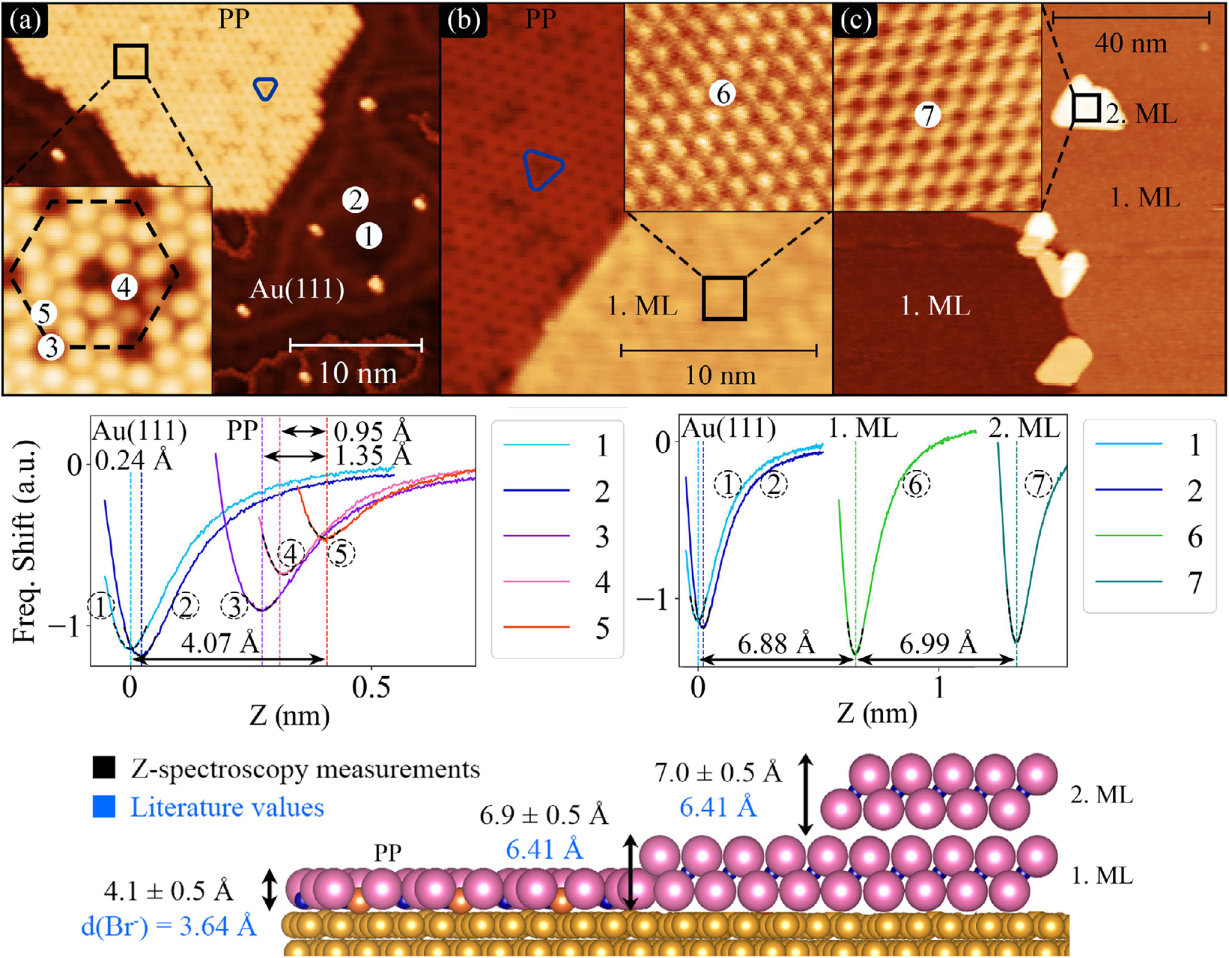
STM images and Z‐spectroscopy of CoBr_2_ a) PP, b) 1. ML and c) 2. ML with Z‐spectroscopy measurement points labeled (1–7). The heights, relative to the off‐herringbone site (1) are: PP =4.1 ± 0.5 Å, 1. ML=6.9 ± 0.5 Å and 2. ML=13.9 ± 0.5 Å (1. ML‐2. ML= 7.0 ± 0.5 Å), respectively. Bottom: schematic side‐view representation with measured and literature values for comparison.

We have analyzed the growth of CoBr_2_ on clean Au(111) substrates via scanning tunneling microscopy (STM) and low‐energy electron diffraction (LEED), by preparing samples ranging from sub‐monolayer to multilayer coverage. In the following, we describe the structural evolution in three stages: i) the precursor phase (PP) observed at sub–monolayer coverages (and discussed in detail later), ii) its reorganization into the bulk‐like monolayer (ML) and iii) the formation of a second monolayer (2. ML) and multilayer films at higher coverages. **Figure** **2** summarizes the main observations. At the lowest coverages, the STM image in Figure 2a reveals that the initial growth phase of CoBr_2_ evenly covers the Au(111) terraces, with domain boundaries clearly visible (blue arrows). We denote this phase as the precursor phase (PP). LEED measurements of this coverage, as well as atomic‐resolution STM images (**Figure** **3**a), reveal a complex diffraction pattern consistent with a surface reconstruction, as we will discuss later. The new diffraction spots observed at low coverage close to the Au main spot (marked with brown circles in the inset of Figure 2a) appear ±5° rotated with respect to the Au(111) substrate's main axes and are associated to the two rotational domains of the PP, consistent with earlier reports for the isostructural first layer of FeBr_2_ on Au(111).^[^ ^21^ ^]^ A more detailed analysis of the domain boundaries and the corresponding LEED pattern is provided in the (Figure S1, Supporting Information).

Upon increasing the coverage (Figure 2b–d), the complex diffraction pattern disappears and is replaced by a hexagonal pattern aligned with the Au(111) diffraction spots (blue and green circles in Figure 2b, respectively). The fact that the diffraction spots of the ML realign with those of Au(111) also indicates that the PP does not remain buried underneath subsequent layers. If the PP were preserved as an underlying rotated structure, the overgrowing layers would inherit its ±5° misalignment with respect to Au(111). Instead, the disappearance of the rotated spots demonstrates that the PP itself transforms into the commensurate ML phase upon increasing coverage. This evidence reflects the stronger epitaxial coupling of the ML to the substrate: while the PP is stabilized as a surface reconstruction with rotated domains, the ML minimizes its energy by adopting the commensurate orientation with Au(111).

Moreover, a closer comparison of the LEED patterns also reveals that the lattice parameter of the ML is smaller than that of the PP. The measured distance ratio (*d*_ML_/*d*_PP_ = 1.046) corresponds to an ≈5% larger lattice constant for the PP (Figure S3, Supporting Information). This is consistent with atomically resolved STM and AFM measurements (Figure 3b,c), where calibration against the clean Au(111) surface also showed a 5% larger lattice constant for the PP (*d*_PP_/*d*_ML_ = 3.9 Å/3.7 Å = 1.054) (Figures S4 and S5, Supporting Information). As we will see later in more detail, DFT calculations support this trend, revealing that under a ±5.21° rotation and average lattice expansion of 5%, a periodic coincidence with Au(111) exists for the PP.

Once the ML is established, further deposition leads to the nucleation of 2. ML islands on top of the 1. ML of CoBr_2_ Figure 2c. STM images clearly reveal, that the growth of the 2. ML islands never takes place on top of the PP (extra evidence of this behavior are shown in Figure S2, Supporting Information). LEED patterns confirm that the 2 ML and subsequent layers preserve the same epitaxial relationship as the ML structure. At even higher coverages, the growth proceeds in a layer–by–layer fashion, giving rise to multilayer films with long‐range order.

To explore the different phases observed by LEED and STM in more detail, we determined the layer height using Z–spectroscopy via non‐contact AFM with a CO–functionalized STM tip.^[^ ^24^, ^25^ ^]^ In this method, the tip is approached vertically toward the surface while recording the frequency shift of the oscillating cantilever.^[^ ^26^ ^]^ The resulting force–distance curves exhibit a characteristic minimum, which corresponds to the equilibrium position between attractive and repulsive tip–sample forces. By comparing the Z‐value of this minimum for different regions of the surface (Au substrate, PP, 1 ML, 2 ML), the relative layer heights can be determined.^[^ ^26^ ^]^

Figure 3d,e shows the Z‐spectroscopy performed on 7 different points, indicated in the atomic STM images Figure 3a–c, together with a schematic representation of the heights for the PP, 1 ML and 2 ML. Our measurements for the first and second ML show a layer height of 6.9 ± 0.5 Å and 7.0 ± 0.5 Å respectively, which agrees with the interlayer distances calculated for the bulk material (6.41 Å in Figure 1)^[^ ^27^ ^]^ and roughly corresponds to twice the Br^−^ anion diameter ($d_{{\mathrm{Br}}^{-}}=3.64$ Å) in an octahedral configuration (6.58 Å).^[^ ^28^ ^]^ This also shows that the strong polarization of CoBr_2_, resulting from the significant difference in electronegativity between the halogen and the transition metal, imparts an almost ionic character to the atoms.

In contrast, the PP exhibits a height of 4.1 ± 0.5 Å measured on top of the protrusions (Figure 3a ‐ Point number (5)), which closely agrees with the diameter of the Br^−^ anion, ruling out the 1T structure, as a possible configuration. This implies that the CoBr_2_ molecules are not directly growing in the bulk‐like structure (Figure 1), but a new phase appears.

Z‐spectroscopy measured on the periodic dark spots (Figure 3a ‐ Point number 3) demonstrates that these correspond to vacancies. We measured a depth of 1.4 Å, at which point repulsive interactions from neighboring Br atoms prevent further penetration of the CO–functionalized tip. Moreover, in our STM images we also observe randomly distributed triangular darker regions, which at atomic resolution appear as three protrusions arranged consistently with the lattice symmetry, but with lower apparent contrast than the surrounding areas (blue triangles in Figure 3a,b). Z‐spectroscopy revels that these atoms are positioned roughly 1.0 Å deeper than the rest of the surface atoms. The most intriguing aspect of these triangular defects is that they exclusively appear in one orientation within a domain, further indicating that the precursor phase is not amorphous but ordered. While AFM measurements reveal a uniform height of the top‐layer Br atoms and identify the hexagonal network of dark spots as vacancies, X‐ray photoelectron spectroscopy (XPS) confirms stoichiometric growth (Figure S6, Supporting Information) with a Co:Br ratio of 1:2. These observations, together with structural models consistent with stoichiometric composition, point toward an arrangement of molecular CoBr_2_ units stabilized on the surface. In this view, the PP corresponds to a molecular layer distinct from the bulk‐like 1T trilayer, which only emerges after reorganization at higher coverages.

These conclusions provide important context for previously reported observations. As was mentioned in the introduction, the same STM and LEED patterns were previously reported for FeBr_2_ on Au(111).^[^ ^21^, ^23^ ^]^ In particular, Hadjadj et al.^[^ ^21^ ^]^ identified an epitaxial reconstructed phase for FeBr_2_ on Au(111) that displays a well–defined LEED pattern and long–range order. However, they refrained from proposing an explicit structural model beyond the epitaxial registry, leaving the atomic–scale arrangement unresolved. More recently, Xiang et al.^[^ ^23^ ^]^ proposed that the reconstructed phase could be described as a defective 1T trilayer containing a high density of Br vacancies, supported by STM/AFM measurements and DFT calculations. While such a model accounts for some features of the reconstruction, it would imply a layer thickness much larger than what we measure, as well as a pronounced deviation from the 1:2 stoichiometry. Both aspects are incompatible with our experimental data: the AFM heights clearly correspond to a single molecular layer and a defect density as high as that proposed should leave a detectable fingerprint in XPS, which has not been seen. Our results thus support a different interpretation, namely that the PP corresponds to a molecularly stabilized arrangement of CoBr_2_ units, that reorganizes into the octahedral 1T ML at higher coverages.

**Figure 4:**
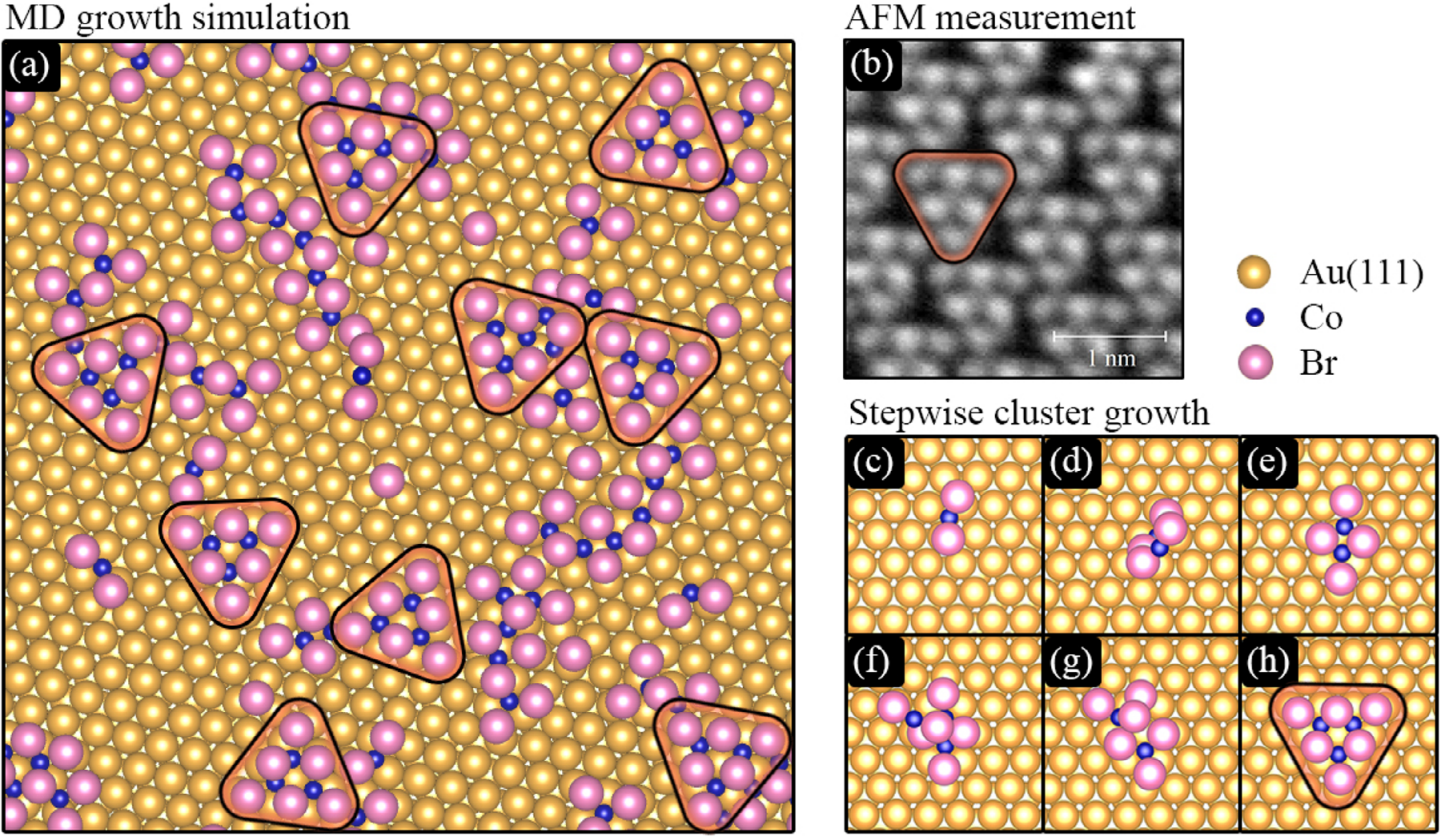
Results of MD simulations of the deposition of 63 CoBr_2_ molecules on an unreconstructed Au(111) surface. a) Intermediate configuration where triangular clusters of three molecules (Co_3_Br_6_) are seen as a recurring motif. b) An AFM image highlighting a similar triangular motif in the PP (see also Figure S9, Supporting Information). c–h) illustrate how the triangular Co_3_Br_6_ cluster is formed through the subsequent arrival of three CoBr_2_ molecules at the Au(111) surface. Note the slight molecular clusters misalignment with the Au(111) lattice vectors that match the experimental observations.

With this in mind, we performed molecular dynamics (MD) simulations using a universal machine–learning interatomic potential trained on a large database of first–principles electronic–structure calculations^[^ ^29^ ^]^ (see Methods). We simulated the deposition of CoBr_2_ molecules at 300 K on an unreconstructed Au(111) surface, allowing 1000 relaxation steps (1000 steps = 2 ps) between each deposited molecule. Given that the interatomic potential is based on first–principles calculations, the only assumptions in our simulations are the structure of the Au(111) substrate and the deposition of a molecule composed of one Co and two Br atoms. No other assumptions were made regarding molecular coordination, structure and arrangement on the surface, or about the interaction mechanisms. The resulting configuration after the deposition of 63 CoBr_2_ molecules, shown in **Figure** **4**, reveals several key insights. The CoBr_2_ molecules, upon reaching the surface, bend to ≈104° and the Co atom always orients toward the gold surface. This highlights the preference of the Au(111) surface to interact with Co atoms rather than with Br atoms of the CoBr_2_ molecule. Furthermore, during deposition, CoBr_2_ molecules on the surface spontaneously form new bonds with new molecules landing on the surface. This process gives rise to molecular aggregation with an almost exclusive three‐fold coordination of Co with Br. A particularly interesting observation is that triangular clusters of three molecules (Co_3_Br_6_) consistently emerge as a recurring motif (triangles in Figure 4), matching the number of molecules within each periodic unit in the PP. The calculations suggest that these clusters are always centered on top of a surface gold atom. Note that the molecular clusters maintain a slight misalignment with the Au(111) lattice vectors, agreeing with the observed rotation found in STM, AFM, and LEED of the PP. It is important to remember, that the relatively short total MD simulation time (200 ps) does not allow us to observe subsequent rearrangements of the molecules at the surface.

**Figure 5:**
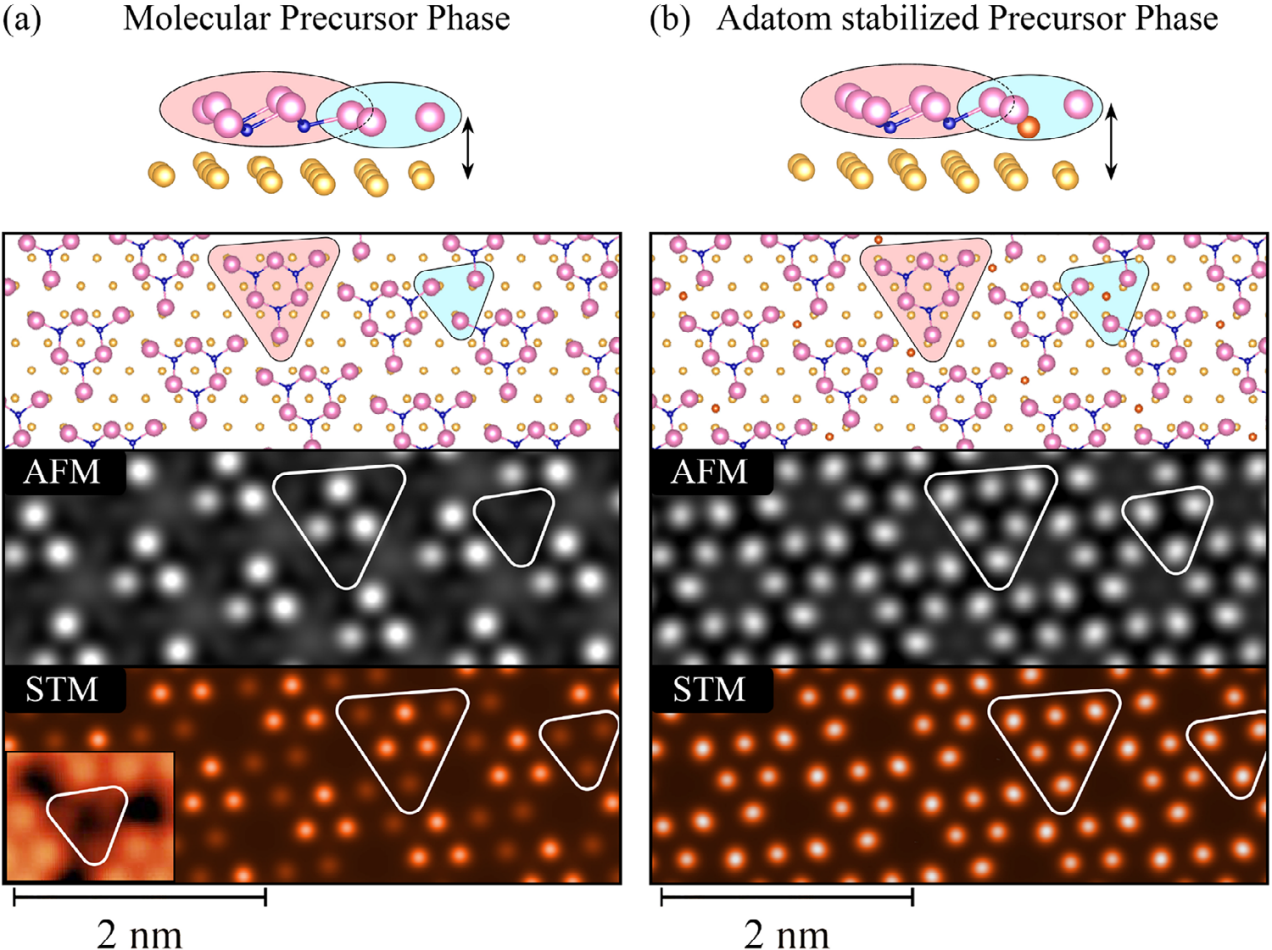
Schematic of a DFT optimized structure a) without and b) with an adatom stabilizing the CoBr_2_ clusters and the corresponding AFM and STM simulations. AFM: the frequency shift is simulated at 4.5 Å above the top layer Br atoms. STM: the electron density is integrated over the energy range from −0.5 eV to Fermi energy *E*_*F*_ at 1.4 Å from the top Br layer. According to the Tersoff–Hamann approximation, this quantity is proportional to the tunneling current at a bias of −0.5 V.

We then carried out a DFT optimization of this recurring motif on Au(111), applying periodic boundary conditions based on the experimentally measured periodicity. The corresponding AFM and STM images, shown in **Figure** **5**a, were simulated based on this optimized structure and display three CoBr_2_ molecules arranged in a cluster. The resulting configuration exhibits a relaxation of the cluster edges toward the surface, leading to three Br atoms positioned at a lower depth. Within a periodic lattice, the arrangement of these clusters naturally aligns in a manner that closely resembles the triangular defects observed in STM/AFM. This configuration also provides a plausible explanation for their exclusive occurrence in one orientation (blue triangles in Figure 3a,b). To prevent a relaxation of the cluster edges toward the surface, not observed in AFM or STM, we stabilized the three‐molecular clusters with an adatom (Figure 5b). In our system, this can happen either via cobalt or via gold due to the large amount required and leads to the formation of an adatom stabilized precursor phase. Both adatoms yielded an atomically flat Br surface in DFT, and the simulated AFM and STM images reproduced the experimental measurements.

However the presence of Au atoms is more likely to occur for several reasons. First, stabilizing the cluster edges with an Au adatom preserves the stoichiometry of the evaporated material, facilitating the transition to the 1T trilayer at higher coverages without altering the elemental composition. Au adatoms are frequently observed in organic molecular assemblies,^[^ ^30^, ^31^, ^32^, ^33^, ^34^ ^]^ where they play a crucial role in stabilizing and guiding molecular organization into ordered nanostructures. Second, we note that the Au(111) herringbone reconstruction, which is a sensitive fingerprint of surface stress relaxation, is not visible beneath the PP or the bulk‐like ML and it is noticeably modified on clean Au(111) regions (Figure 3a). This behavior contrasts with that of transition metal dichlorides, where the herringbone reconstruction remains visible beneath the 1. ML.^[^ ^22^, ^35^ ^]^ This indicates a stronger substrate interaction for transition metal dibromides and supports our interpretation that Au adatoms actively contribute to the stabilization of the PP.

Moreover, the amount of triangular defects depends on the substrate temperature during CoBr_2_ deposition. At lower substrate temperatures during growth, a larger number of triangular defects is observed (Figure S13, Supporting Information). This trend is consistent with the findings of Xiang et al.,^[^ ^23^ ^]^ who also reported a reduction in triangular defects with increasing growth temperature. A higher temperature facilitates the mobility of Au atoms, thereby reducing the formation of triangular defects during growth.

A stabilizing Co adatom instead of Au, would require a decomposition of CoBr_2_, leading to a change in stoichiometry (Co_4_Br_6_) within the PP. We observe a small amount of Br adsorbed on the pristine gold regions forming meshes (Figure S11, Supporting Information).^[^ ^36^, ^37^ ^]^ Although their origin is unclear, these Br meshes could indicate an excess of Co atoms, leading to the formation of non‐stoichiometric Co_4_Br_6_ clusters. Upon monolayer formation at higher coverages, where the Co to Br ratio is 1:2, there would exist an excessive metallic Co atom due to the reaction Co_4_Br_6_ →  3 CoBr_2_ + *Co*. Even though we did not observe metallic Co in XPS or desorption experiments, we cannot exclude this possibility (further discussion about a non‐stoichiometric PP and the corresponding XPS spectra can be found in, Figures S6 and S7, Supporting Information).

**Figure 6:**
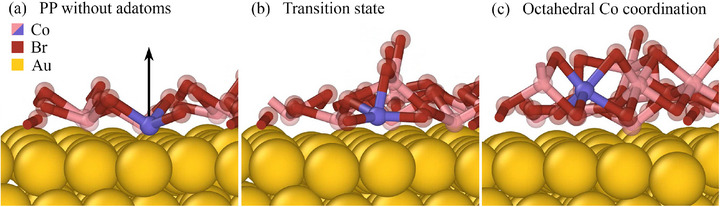
CoBr_2_ growth simulated at 300 K over a time period of 1 ns starting from the PP configuration without adatoms a) (Figure 5a). An increase in coverage, leads to a reduction of the available space per molecule and to an increase in cobalt's coordination number (= 3 in PP; 6 in ML). At some positions, a six‐fold coordination resembling the octahedral 1T trilayer structure found in the bulk material evolves over the course of the simulation. The respective Co atom is highlighted in blue. Note also the evolution of the Br atoms, which transition from being positioned above the Co layer to moving beneath it, coming into direct contact with the gold as the coverage increases.

To gain further microscopic insight into the transformation from the PP to the ML, we performed MD simulations at 300 K, starting from a fully covered PP structure (as in Figure 5a) and progressively adding additional CoBr_2_ molecules to mimic continued deposition. Representative snapshots of the process are shown in **Figure** **6**. In the initial PP configuration Figure 6a, the Co atoms lie in direct contact with the Au surface (one is highlighted in blue in Figure 6). Upon increasing coverage Figure 6b, the newly deposited molecules drive a gradual rearrangement of the interfacial atoms: Co atoms are lifted from the substrate, while Br atoms shift downward to occupy positions closer to the gold surface, giving rise to the octahedral coordination characteristic of the 1T monolayer (Figure 6c). Although the accessible timescales of the simulation are limited to a few picoseconds, the results clearly capture the onset of the structural transformation and illustrate the mechanism by which the PP reorganizes into the ML. We note that the present model does not yet include adatoms explicitly, but the observed rearrangement provides strong microscopic support for our experimental interpretation.

This simulated transition supports the experimental observation that the complex LEED pattern of the PP disappears as the bulk–like ML is established, together with the alignment of the diffraction spots to those of Au(111). Importantly, the STM data at intermediate coverages (Figure 2c; Figure S2, Supporting Information) show that 2. ML islands nucleate only on top of the 1. ML and never on the PP, indicating that the PP does not act as a template for further growth but rather reorganizes into the ML itself. Together, these findings demonstrate that the PP is not buried beneath subsequent layers, but instead reorganizes into the epitaxial ML. Additionally, MD simulations confirm that once the 1T ML is formed, it is structurally robust and remains stable up to 500 K, where desorption takes place directly, without any preceding reorganization. This high thermal stability further excludes the possibility that the PP corresponds to a defective–vacancy trilayer,^[^ ^23^ ^]^ as such a non‐stoichiometric structure would neither reorganize cleanly into the ML nor yield the experimentally observed long‐range, thermal stability of the 1T structure.

## 3 Conclusion

In this study, we have shown that a molecular precursor phase (PP) precedes the growth of the bulk–like van der Waals crystal layer structure of CoBr_2_ on Au(111). Our findings point to a two‐step growth mechanism where the CoBr_2_ molecules initially lie flat on the surface exhibiting a well–defined periodicity with a hexagonal arrangement of Br atoms and a characteristic network of holes. These features arise from a structural coincidence with the substrate, under a ±5° rotation relative to the Au(111) lattice. The combined experimental evidence and theoretical calculations indicate that Au adatoms are the primary species responsible for stabilizing the PP phase. Since XPS measurements confirm stoichiometric growth of CoBr_2_, the involvement of a significant number of detached Co atoms (or other atomic species) in stabilizing the PP can be excluded.

Upon reaching a critical coverage, the PP rearranges into a 1T trilayer isostructural to the bulk material, emphasizing the role of intermediate phases in the formation of vdW thin films and hinting to a coverage–driven structural rearrangement. Structurally, the PP unit cell contains three CoBr_2_ molecules, whereas the equivalent cell in the bulk–like ML contains seven, implying a significantly higher packing density in the ML. Combined with our molecular dynamics simulations, it shows that the PP reorganizes into the denser octahedral ML, rather than persisting as a buried layer.

Our results highlight the effectiveness of machine–learning–driven MD simulations, combined with direct DFT calculations, in providing insights into atomic‐scale growth behavior of 2D materials. These simulations identified a recurring molecular motif, elucidated the preferred Co–Au over Br–Au interaction upon adsorption, revealed the slight misalignment with the substrate and confirmed the stability of this motif in a periodic lattice, all in agreement with experimental data.

More broadly, our findings indicate the existence of a surface‐stabilized precursor phase preceding the growth of the van der Waals layers. The identification of such a precursor phase challenges the conventional view of direct monolayer growth and suggests that similar mechanisms may govern the formation of other transition metal dihalides.

## Conflict of Interest

The authors declare no conflict of interest.

## Supporting information

Supporting Information

## Data Availability

The data that support the findings of this study are available in the supplementary material of this article.
