# Supplementary material for: Coverage–Dependent Structural Evolution of CoBr2 at the Au(111) Interface
Source: Adv Sci (Weinh). 2025 Nov 14;12(47):e08262. doi: 10.1002/advs.202508262 (PMC12713089; doi:10.1002/advs.202508262)
Supplement: Supplementary file 1 — Supporting Information [file ADVS-12-e08262-s001.pdf]

Coverage-Dependent Structural Evolution of $CoBr_2$ at the Au(111) Interface

Samuel Kerschbaumer [*,†] Martin Ondráček [‡] Sebastien E. Hadjadj [†] Oleksandr Stetsovych [‡] Andrés Pinar Solé [‡] Adriana Elizabet Candia [†,¶] Paula Angulo-Portugal [†] Andrea Aguirre-Baños [†] Martina Corso [†] David Serrate [§,‖] Jorge Lobo-Checa [§,‖] Pavel Jelínek [‡] Maxim Ilyn [†] Pablo M. Piaggi [*,⊥,#] and Celia Rogero [*,†]

†*Centro de Física de Materiales (CSIC/UPV-EHU), 20018 Donostia-San Sebastián, Spain*

‡*FZU - Institute of Physics of the Czech Academy of Sciences, Cukrovarnická 10, Prague 6, CZ 16200, Czech Republic*

¶*Laboratorio de Microscopias Avanzadas (LMA), Universidad de Zaragoza, Zaragoza E-50018 Spain*

§*Instituto de Nanociencia y Materiales de Aragón (INMA), CSIC-Universidad de Zaragoza, 50009 Zaragoza, Spain*

‖*Departamento de Física de la Materia Condensada, Universidad de Zaragoza, E-50009 Zaragoza, Spain*

⊥*CIC nanoGUNE-BRTA, Tolosa Hiribidea, 76, Donostia-San Sebastián, 20018, Spain*

#*Ikerbasque, Basque Foundation for Science, 48013 Bilbao, Spain*

E-mail: kerschbaumersamuel@gmail.com; pm.piaggi@nanogune.eu; celia.rogero@ehu.eus

Supplementary Information

Experimental

Cobalt dibromide ($CoBr_2$) films (sub-monolayer to multilayer), were grown on Au(111) by thermal evaporation of ultra pure molecular powder from a Knudsen cell evaporator with quartz crucibles (Dodecon OMBE Source[1]). The evaporation was performed under ultra high vacuum (UHV) conditions (evaporation pressure of 10^{-8} mbar to 10^{-9} mbar) at a temperature of roughly 400°C. The purity of the anhydrous beads of $CoBr_2$ from Sigma Aldrich used in the experiment was labeled at 99.9 %[2] and the substrate cleaning process involved standard Ar^+ sputtering and annealing cycles at 720 K. Several attempts were made to optimize the Au(111) substrate temperature for deposition. Within the range of room temperature to 180°C, $CoBr_2$ can be reliably grown, consistently yielding well-ordered structures. Below room temperature, the material fails to form an ordered phase, while at 200°C, it does not stick to the substrate. This is also observed for $FeBr_2$ on Au(111) by Hadjadj et. al.[3] The amount of evaporated material was estimated via a quartz microbalance, while low-temperature scanning tunneling microscopy (LT-STM), X-ray photoelectron spectroscopy (XPS) and low-energy electron diffraction (LEED) were used as cross-reference. The average evaporation time for all samples was between 1-5 min.

The STM experiments were performed with a commercial Scienta-Omicron LT-STM at 4.3 K and 10^{-10} mbar base pressure. The AFM experiments were performed with a SPECS Low-temperature Scanning Probe Microscope with Joule-Thompson stage at 1.4 K and a Kolibrí AFM force sensor with W tips functionalized in UHV with a carbon monoxide -CO molecule.[4] All AFM measurements were performed at constant height. The base pressure was 10^{-10} mbar and Nanonis software was used. The resonance frequency of Kolibri sensor is ~990000 Hz and the oscillation amplitude we use for all measurements is 50 pm. Regarding, the Z-spectroscopy height measurements, since the growth of CoBr2 does not allow the

coexistence of clean Au(111) and first ML regions, the height of the first ML was measured relative to the PP. The height of the second ML was determined with respect to the first ML. The XPS measurements were carried out with a Phoibos 100 photoelectron spectrometer, using a non-monochromatic Al-Kα X-ray source. The energy resolution is 0.1 eV. UHV conditions were preserved during all the sample transfers (base pressure during experiment was 10^{-10} mbar).

MD simulations were performed using the Atomic Simulation Environment 3.23.0.[5] A 2-fs timestep was used for the integration of the equations of motion, and the temperature was controlled using a Langevin thermostat with a damp parameter of 1 ps and a target temperature of 300 K. The interatomic interactions were described using the MACE-MP *medium* foundation model[6] with D3 dispersion correction.[7] This model is trained on a very general database of the Materials Project. We validated this model by simulating the ML and PP structure over the Au(111) layer, as well as a clean Au(111) surface, and we observed that all such structures were stable over a 1-ns-long MD run. This shows that the MACE-MP model is able to describe the two limiting structures (ML and PP) known to be of relevance from experiments and direct DFT calculations. The MACE-MP model is trained on approx. 1.5 million configurations of the Materials Project,[8] including the ML structure of $CoBr_2$ and more than 100 other crystal structures that include Au, Co, and/or Br atoms. The simulation box with the Au(111) slab was defined through two vectors with lengths of 62 Å and 54 Å which formed an angle of 125° in the plane of the surface, and had a length of 60 Å in the direction perpendicular to the surface. The slab had a total of 1872 Au atoms (four Au layers) and the Au atoms in the bottom-most layer were kept fixed throughout the simulation. Linear $CoBr_2$ molecules, with a Co-Br distance of 2.3 Å were created every 1000 MD steps at around 40 Å above the surface with an initial velocity in the -z direction, i.e. towards the Au(111) surface. A total of 108 molecules were deposited on the surface using this protocol.

Geometry optimization of the proposed structures was done with the plane-wave DFT

package VASP[9,10] using the PBE[11] generalized-gradient approximation for the exchange-correlation functional. Each of the model slabs consisted of 4 atomic layers of the Au(111) substrate, the specific $CoBr_2$-derived structure on top of it and about 19 Å (for the PP) or 17 Å (for the ML) of vacuum space, making the total thickness of the slab cell 30 Å. The plane-wave basis set was defined by the kinetic energy cutoff 400 eV. All grids to represent potentials and densities were defined by this basis-set cutoff and the precision option set to `Accurate`. The Co, Br, and Au elements were represented by standard projector-augmented wave (PAW) potentials.[12] The convergence criterion for electron self-consistency was set as the total-energy change not exceeding 1 meV; the optimal geometry was taken to be achieved when forces on all atoms dropped under 0.01 eV/Å. The unit cell sizes were rigid, defined by the empirical lattice constant of Au. The surface Brillouin zone was sampled with a 6×6 k-point mesh. All the DFT calculations were performed as spin-polarized, with the initial estimate of magnetic moments 2.0 μ_B for the Co atoms (and 0 for Br and Au atoms). Van der Waals dispersive forces were estimated with the Tkatchenko-Scheffler method.[13]

The AFM images were simulated using the `ppafm` package,[14,15] taking the DFT-calculated electron density of the structures as an input. The electron density of the AFM tip, used to calculate the repulsive forces, was taken to be that of a CO molecule. The attractive part of the force was approximated as a van der Waals force calculated according to the DFT-D3 method.[7] Electric charges or multipoles of the tip were disregarded. The lateral mechanical stiffness of the AFM tip termination (effectively the O atom of the CO molecule) was assumed to be 0.25 N/m; the radial stiffness 20 N/m.

The STM images were simulated in the Tersoff-Hamann approximation using the standard tool available in the FHI-aims DFT code.[16,17] The FHI-aims utilizes an atom-centered basis set. The suggested `tight` basis sets, provided with the code, were employed in the STM simulations for all elements. For the sake of consistency, geometries optimized with the FHI-AIMS code (rather than VASP) were used for STM; however, only the `light` (smaller) default basis set was used during the geometry relaxation.

Rotational Domains of the PP

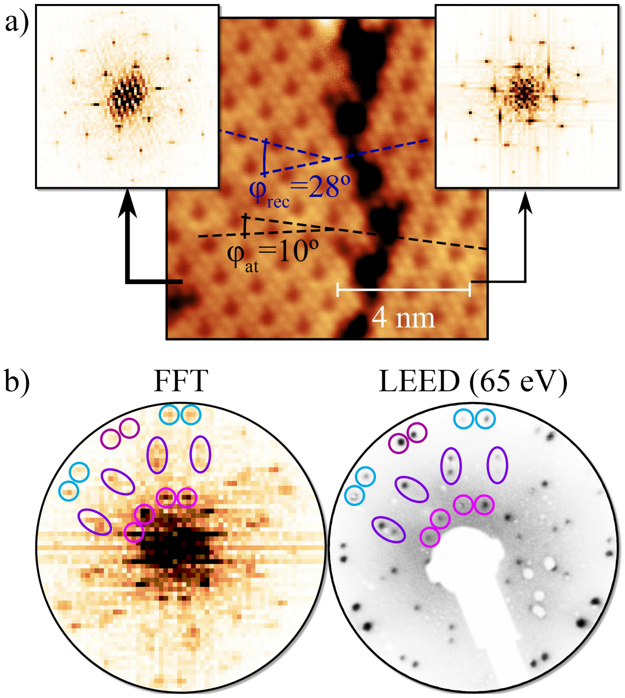

Figure S1: a) The STM image of two rotational domains of the PP of $CoBr_2$ shows atomic rows, denoted as φ_{at}, that are rotated by 10° relative to each other (marked by a black dashed line). The lattice vectors of the reconstruction in these domains, φ_{rec}, exhibits a rotation of 28° (highlighted by a blue dashed line). Fast Fourier transforms (FFT) of the individual terraces are displayed on either side of the STM image. b) Below, the FFT of the entire STM image and a LEED pattern recorded at 65 eV is presented. The light blue circles indicate the diffraction spots of $CoBr_2$, arising from the periodic spacing between neighboring Br atoms. The violet and purple spots correspond to the first- and second-order diffraction features of the reconstruction, defined by the periodic arrangement of holes, demonstrating that all diffraction spots observed in the STM image are accurately reproduced by both experimental techniques.

Figure S1 (a) shows an atomic resolution image of the two PP rotational domains at their boundary, along with their respective Fast Fourier Transforms (FFTs). Each rotational domain exhibits a hexagonal pattern from the atomic arrangement and a superstructure appearing as a triangular network of dark spots (= holes). Combining the FFTs of both rotational domains yields the pattern in (b), which was directly calculated from the STM image and reproduces all diffraction spots visible in LEED at 65eV. Further analysis reveals that the angle between the close-packed directions of Au(111) and the topmost Br plane

is 5.3°, while the angle between the high-symmetry directions of Au(111) and the lattice vectors of the superstructure is about 13.8°. According to Hadjadj et al.,[3] in the case of $FeBr_2$ (which is isostructural in STM), this 5.3° rotation of the Br plane leads to a periodic coincidence with the underlying Au(111) substrate.

PP vs ML on large scale STM images

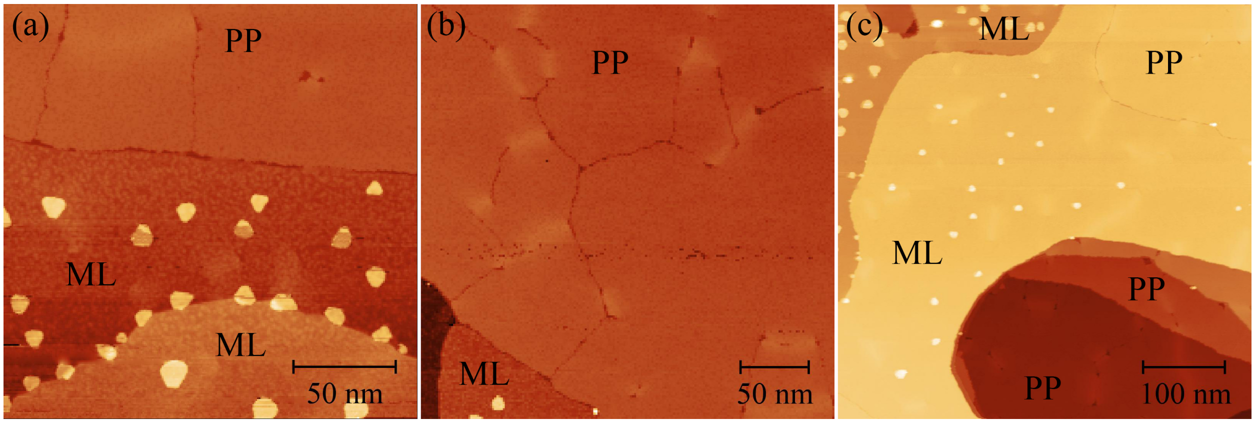

Figure S2: $CoBr_2$ in the 1–2 ML coverage regime exhibits two distinct phases. One phase is characterized by domain boundaries, which are attributed to the PP, while the other consists of small, well-aligned hexagonal second ML islands growing atop the first ML (yellow hexagons). STM parameters: (a) $U = 100$ mV, $I_t = 10$ pA; (b) $U = 100$ mV, $I_t = 10$ pA; (c) $U = 100$ mV, $I_t = 10$ pA.

Figure S2 highlights the distinct chemical environments of the PP and ML. The difference is evident in the formation of islands, which frequently appear on top of the ML structure, whereas the PP remains easily identifiable even at this scale due to its characteristic domain boundaries. This distinction persists at a broader scale of 500×500 nm, further emphasizing the unique chemical nature of each phase. Notably, the second ML islands that form on the first ML exhibit a well-defined hexagonal shape and a consistent alignment. This growth behavior aligns with that of bulk $CoBr_2$, where the single layers stack analogous.[18]

PP vs. ML Distance Ratio - LEED

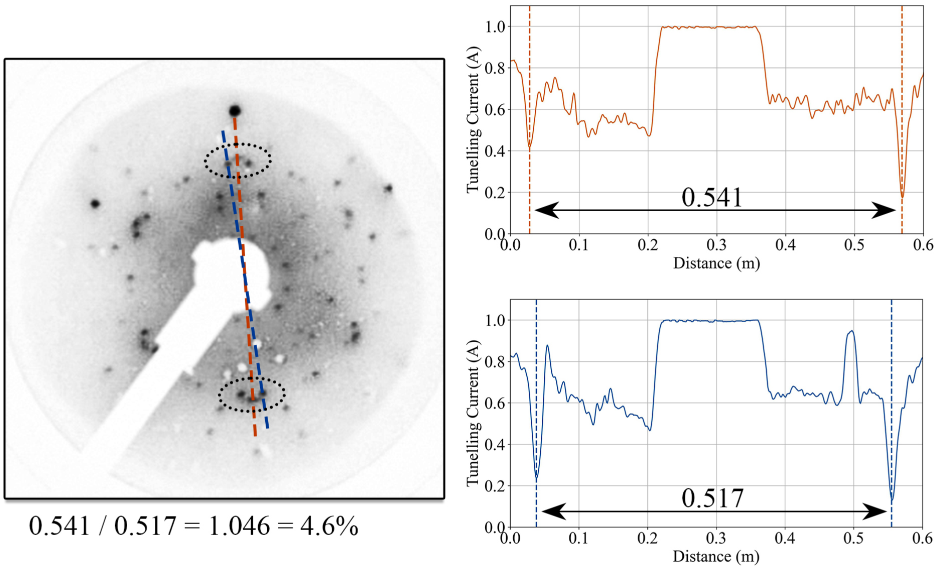

Figure S3: $f = 0.541/0.517 = 1.046 = 4.6\%$. The lattice constant of the PP is 4.6% larger than that of the ML.

SPECS LT-SPM Calibration - AFM

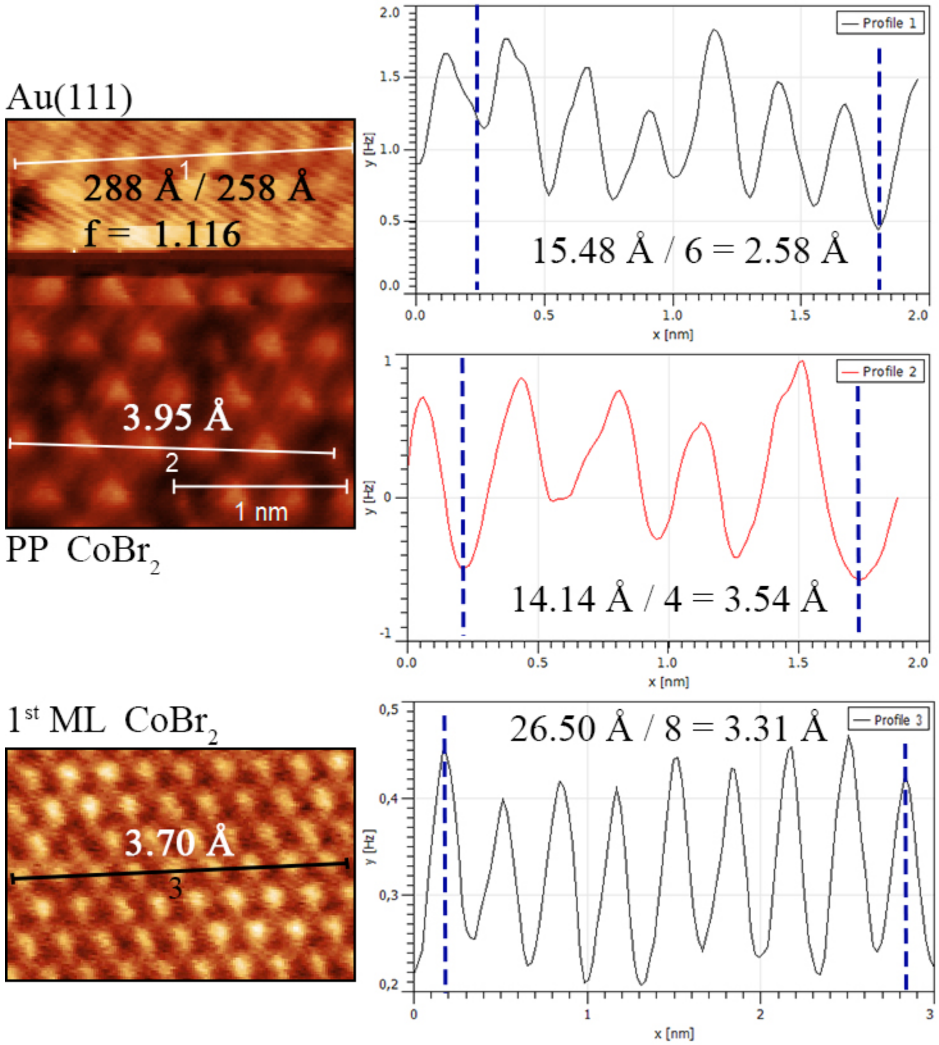

Figure S4: The AFM calibration factor is determined at f = 2.88 Å/2.58 Å = 1.12. The lattice constant of the PP is 3.5 Å $\cdot$ 1.12 = 4.0 $\pm$ 0.1 Å. The literature lattice constant for ML $CoBr_2$ is 3.73 Å.[8] The lattice constant of the PP is 5.9% larger than in the ML. Atomic resolution of Au and $CoBr_2$ is achieved within the same AFM measurement by optimizing the tip-sample distance for both terraces beforehand and manually changing the scan parameters while scanning across the step edge.

Scienta-Omicron LT-STM Calibration - STM

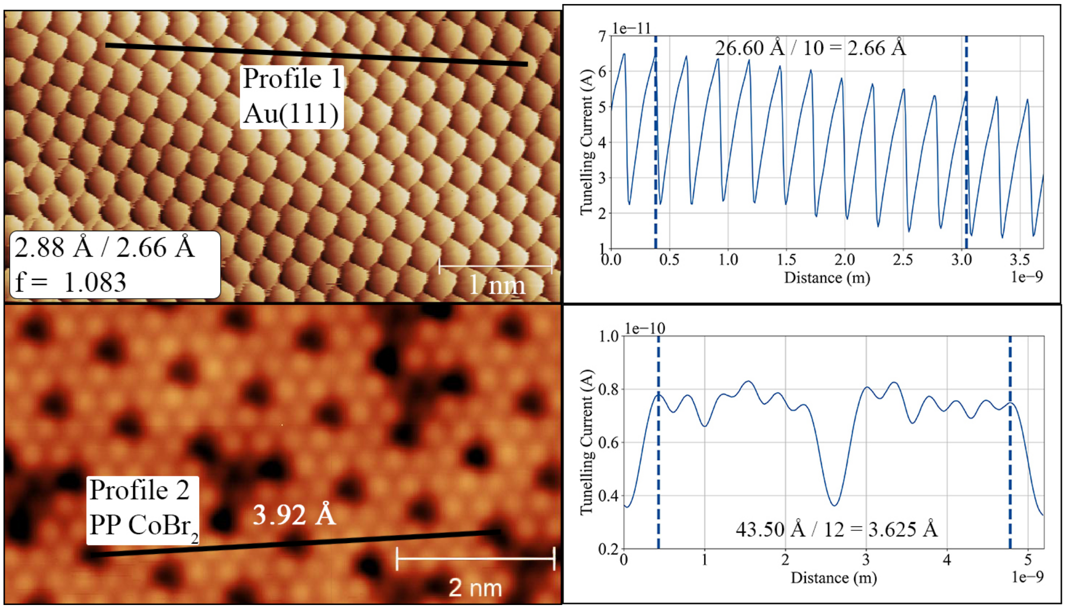

Figure S5: The STM calibration factor is determined at f = 2.88 Å/2.66 Å = 1.08. The lattice constant of the PP is 3.6 Å $\cdot$ 1.08 = 3.9 $\pm$ 0.1 Å. The literature lattice constant for ML $CoBr_2$ is 3.73 Å.[8] The lattice constant of the PP is 5.1% larger than in the ML.

XPS - Co2p, Br3p and Au4d

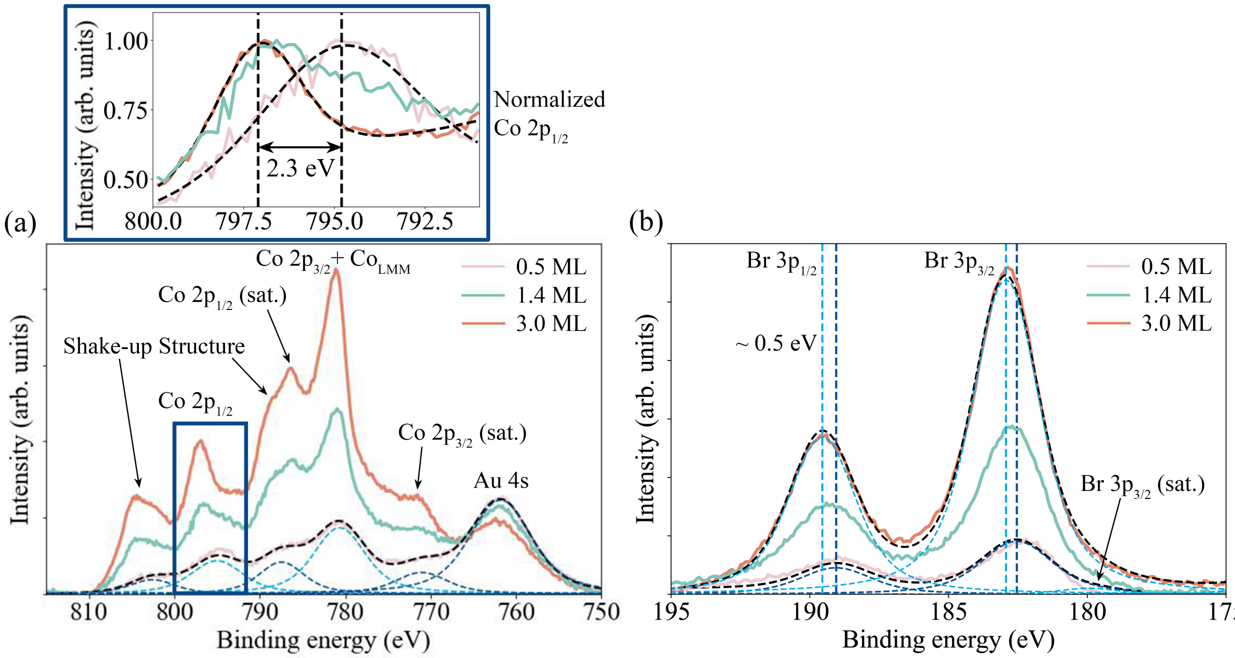

Figure S6: XPS measurements of the Co 2p and Br 3p regions were performed using an Al Kα X-ray source. Across the coverage range from sub-monolayer to multilayer, both regions exhibit a shift toward higher binding energies. The inset focuses on the Co $2p_{1/2}$ region, normalized to enhance the visibility of this shift.

Figure S6, shows the Co 2p and Br 3p XPS spectra for three different coverages of $CoBr_2$ on Au(111). At higher coverages, the spectrum exhibits the characteristic shake-up structure associated with cobalt in the 2+ oxidation state.[19] At lower coverages, both the Br 3p and Co 2p peaks shift slightly toward lower binding energies. This shift can be attributed to the direct interaction between Co atoms and the Au surface, influenced by the electron-donating nature of gold. In contrast, in the bulk-like structure, the Br layer is in direct contact with the Au surface, preventing this effect.

Correctly fitting of the Co 2*p* region is challenging due to the presence of multiple overlapping features. Within this spectral range, at least four shake-up peaks, the primary Co $2p_{1/2}$ and Co $2p_{3/2}$ peaks and the Au 4*s* peak contribute to the complexity. Additionally, the non-monochromaticity of the X-ray source introduces a 10% intensity satellite peak for each core-level feature, further complicating the deconvolution process. The presence of

the Co_{LMM} Auger peak adds another layer of spectral congestion. As a result, achieving a precise fit to extract an exact Co-to-Br ratio from XPS data proved impractical due to the extensive peak overlap and inherent uncertainties in the fitting procedure.

Table S1: XPS Fitting Parameters for $CoBr_2$ on Au(111).

Coverage	Peak	FWHM	Peak Center (eV)
0.5 ML	Br $3p_{1/2}$	3.10	189.06
	Br $3p_{3/2}$	3.10	182.55
	Br $3p_{1/2}$ sat	1.13	179.46
	Br $3p_{3/2}$ sat	1.13	172.95
	shake up	6.48	802.21
	Co $2p_{1/2}$	6.07	795.19
	shake up	6.48	790.00
	shake up	5.96	786.09
	Co $2p_{3/2}$	6.07	780.28
	Co sat.	6.48	770.68
	Au4s	6.48	761.88
	Co_{LMM}	1.45	774.60
	Au4d 3/2	5.46	353.39
	Au4d 5/2	5.46	335.38
	Au4d 5/2 sat	6.48	325.78
	Au4d 3/2 sat	6.48	343.79
1.4 ML	Br $3p_{1/2}$	3.10	189.06
	Br $3p_{3/2}$	3.10	182.55
	Br $3p_{1/2}$ sat	1.13	179.46
	Br $3p_{3/2}$ sat	1.13	172.95
	shake up	0.01	804.97
	shake up	6.48	802.21
	Co $2p_{1/2}$	6.07	795.19
	shake up	6.48	790.00
	shake up	5.96	786.09
	Co $2p_{3/2}$	6.07	780.28
	Co sat.	6.48	770.68
	Au4s	6.48	761.88
	Co_{LMM}	1.45	774.60
	Au4d 3/2	5.46	353.39
	Au4d 5/2	5.46	335.38
	Au4d 5/2 sat	6.48	325.78
	Au4d 3/2 sat	6.48	343.79

Coverage	Peak	FWHM	Peak Center (eV)
3 ML	Br $3p_{1/2}$	2.92	189.56
	Br $3p_{3/2}$	2.92	182.94
	Br $3p_{1/2}$ sat	2.49	179.96
	Br $3p_{3/2}$ sat	2.49	173.34
	shake up	3.55	804.96
	shake up	4.06	802.11
	Co $2p_{1/2}$	4.29	796.85
	shake up	6.48	790.00
	shake up	5.87	786.35
	Co $2p_{3/2}$	4.29	781.19
	Co sat.	6.48	771.59
	Au4s	6.48	762.09
	Co_{LMM}	4.09	776.00
	Au4d 3/2	5.17	353.34
	Au4d 5/2	5.17	335.27
	Au4d 5/2 sat	6.48	325.67
	Au4d 3/2 sat	6.48	343.74

Co_4Br_6 - Non Stoichiometric PP

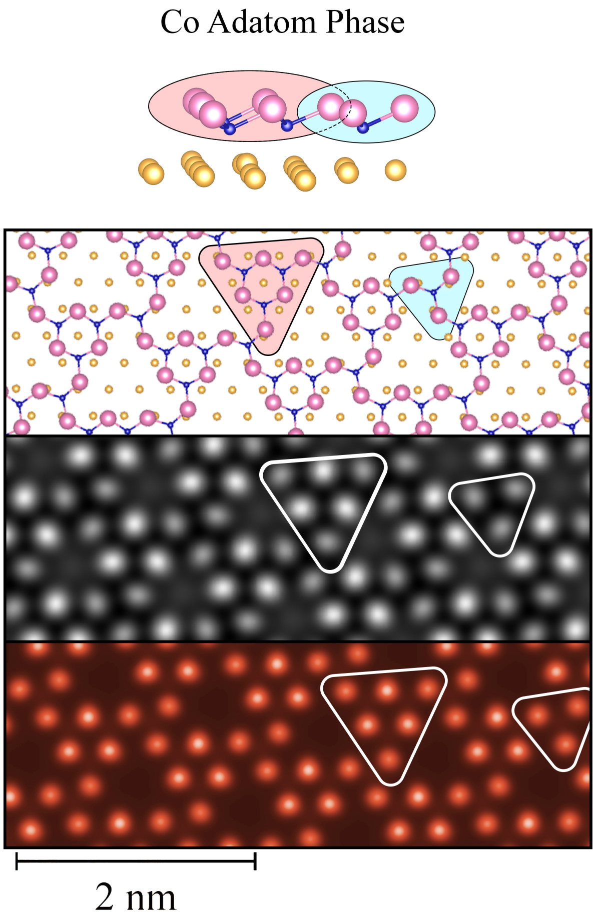

Figure S7: Schematic of a DFT optimized structure with Co adatom stabilizing the $CoBr_2$ clusters and the corresponding AFM and STM simulations. AFM: the frequency shift is simulated at 4.5 Å above the top layer Br atoms. STM: the electron density is integrated over the energy range from -0.5 eV $- E_F$ eV. According to the Tersoff-Hamann approximation, this quantity is proportional to the tunneling current at a bias of -0.5 V.

A possible non-stoichiometric arrangement of the PP features a Co-to-Br ratio of 2:3, which could result from the partial decomposition of $CoBr_2$ on the Au(111) surface. This imbalance could lead to the formation of the structure depicted in Figure S7. Due to the high uncertainty in XPS measurements, we cannot entirely rule out this configuration as a potential PP arrangement. However, several factors make this structure less probable.

First, the formation mechanism of the PP must be considered. A significant stoichiometric imbalance would require substantial decomposition of $CoBr_2$, leading to the presence of

intermediate adsorbed Co and Br species. If this were the case, a considerable number of free Br atoms should either be visible in STM/AFM on the surface or an increased Co content should be detectable in XPS. None of these observations have been sufficiently pronounced to support this interpretation. Temperature-programmed desorption (TPD) studies of Br_2 on Au(100) showed that desorption occurs only above 400 K for coverages exceeding 0.5 ML, conditions significantly different from those in our experiments.[20] The observed amount of free Br on the surface does not align with what would be necessary to create the degree of stoichiometric imbalance proposed for this PP structure.

Second, the fate of the additional Co atom upon transformation into the bulk-like phase raises critical questions. If this proposed non-stoichiometric PP rearranges into a 1T trilayer, the excess Co atoms would need to be accounted for, particularly regarding their oxidation state. In the PP, a fractional oxidation state could be stabilized by interactions with the gold substrate. However, this reaction would leave behind one metallic Co atom per unit cell, as the Co atoms in the bulk-like structure are in a 2+ oxidation state. This would result in 25% of all Co atoms being metallic upon transformation into the ML. Given also that the ML exhibits no observable defects, an initial non-stoichiometric imbalance appears even less plausible.

Furthermore, any remaining fourth Co atom should be detectable in XPS as a metallic species persisting on the surface after temperature-programmed desorption at 200 °C, which has not been observed. The absence of such signatures in our measurements provides further indications against the non-stoichiometric PP configuration.

Atomic resolution AFM and STM of PP, first and second ML

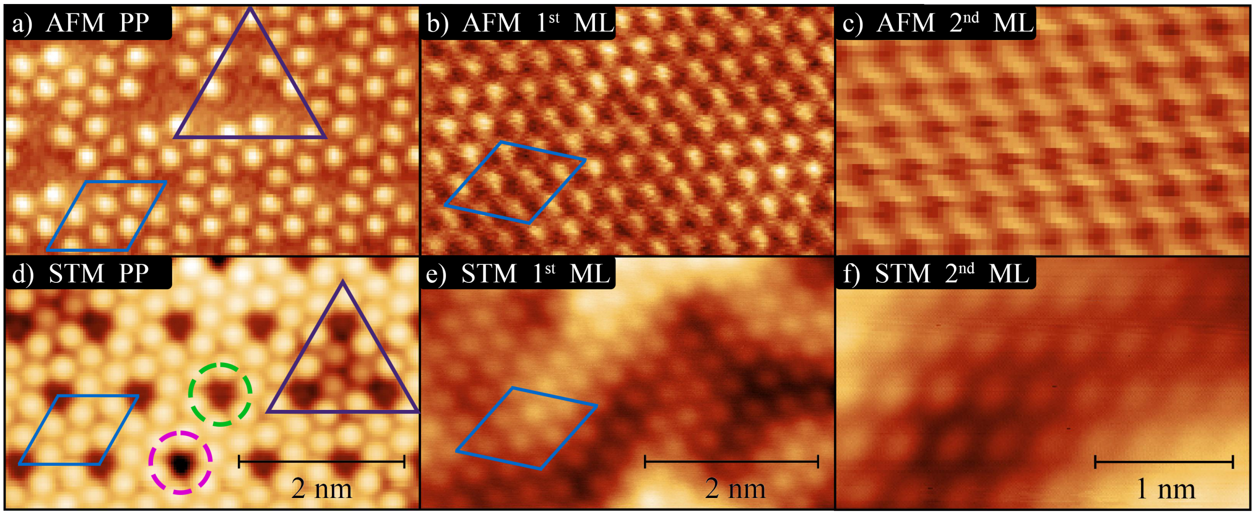

Figure S8: Atomically resolved STM and AFM images. (a) AFM: $U = 10$ mV, $I_t = 10$ pA. (d) STM: $U = 5$ mV, $I_t = 500$ pA. Atomic resolution of the PP reconstruction. The light blue rhombus indicates the unit cell of the reconstruction, which is rotated by 14° compared to (b) and (e). The position marked by the violet triangle indicates a disruption in the periodicity of the PP. Notably, within a single domain, the triangles consistently align in the same direction. (b) AFM: $U = 100$ mV, $I_t = 10$ pA. (e) STM: $U = 200$ mV, $I_t = 40$ pA. 1st ML with a large-scale electronic corrugation exceeding the atomic resolution. This feature is not visible in AFM, indicating a predominantly electronic origin rather than a structural one. (c) AFM: $U = 51$ mV, $I_t = 10$ pA. (f) STM: $U = 800$ mV, $I_t = 13$ pA. 2nd ML exhibits similar behavior to the 1st ML. Note that in (f), creep and drift have caused misalignment of the rows.

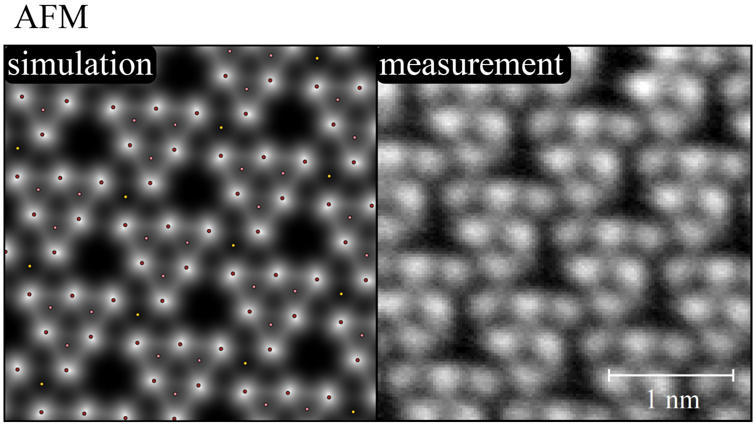

Figure S9: (a) AFM simulation at 4.1 Å above top layer Br atoms and (b) AFM measurement at a reduced tip-sample distance.

While STM imaging reveals the electronic structure of a surface, AFM provides topographic information. The unit cell of the reconstruction is highlighted in blue. Three distinct features are visible in the STM image of the PP: Triangular contrast depletions appearing exclusively on one side of the unit cell and two types of holes, marked by green and purple dashed circles. The green circled contrast depletion is confirmed as a vacancy in Z-spectroscopy, where a penetration of 1.5 Å can be reached, whereas the second (purple circle) proved more difficult to characterize. In the following we briefly outline the experimental procedure that is necessary to differentiate the contrast depletions marked with green and pink circles (Figure S8 (d)). The green and pink circled contrast depletions were first examined in STM under different tunneling conditions. While the STM images appeared identical across the full bias range, features such as the triangular defects and the pink-circled contrast depletion (Figure S8) became clearly distinguishable at biases above 2 V (Figure S10). The same area was subsequently imaged in AFM mode at a very small tip–sample distance, allowing resolution of the three deeper atoms inside the triangular defects as well as the adatom within the vacancy (pink-circled contrast depletion in Figure S8). Z-spectroscopy at the three marked locations showed that positions 1 and 2 were equivalent, whereas position 3 (a "real" vacancy) appeared deeper. Although the quality of these measurements is insufficient for publication in the main text, they clearly indicate the presence of an adsorbate within the pink-circled vacancy.

Both the first and second ML in Figure S8 exhibit a well-ordered hexagonal network of Br atoms, free of defects, consistent with the expected bulk structure. Furthermore, a large-scale electronic corrugation is observed without apparent periodicity. In previous studies on $CoCl_2$ on Au(111) we have attributed similar observations to interface states arising from interactions with the gold substrate. Another possibility is that this large scale corrugation might be related to the adatoms underneath the layer.

When we first observed the green and pink circled contrast depletions in STM (in Figure S8), we investigated their appearance under different tunneling conditions. While the STM

image appears identical across the entire bias range, features such as the triangular defects and the pink circled contrast depletion become clearly distinguishable above 2 V (see Figure S10). Once we had identified the positions in STM, we remeasured the same area with a very small tip-sample distance and were able to resolve the 3 deeper laying atoms inside the triangular defects as well as the adatom inside the vacancy (pink circled contrast depletion in S8). Z-spectroscopy at the three marked locations shows how position 1 and 2 are equivalent, while position 3 (a "real" vacancy) appears deeper. These measurements clearly show the presence of an adsorbate within the pink circled vacancy.

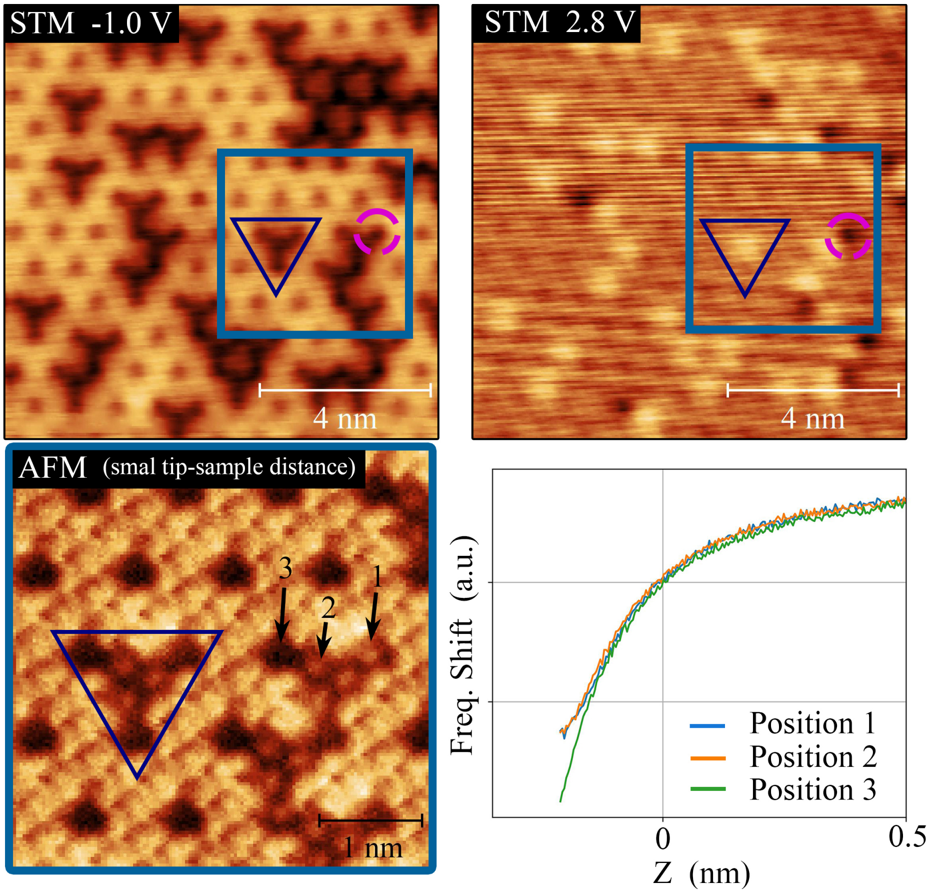

Figure S10: STM images of the same area at -1.0 V and 2.8 V show that the triangular defects (blue triangle) and the pink contrast depletions (also shown in Fig. S8) are clearly distinguishable at both bias voltages. The AFM image presents a section of the surface indicated by the cyan square. Notably, position 1 appears “filled,” which is also evident in the Z-spectroscopy data for the three marked positions: positions 1 and 2 exhibit the same apparent depth, whereas position 3 appears deeper.

Br-mesh on Au(111)

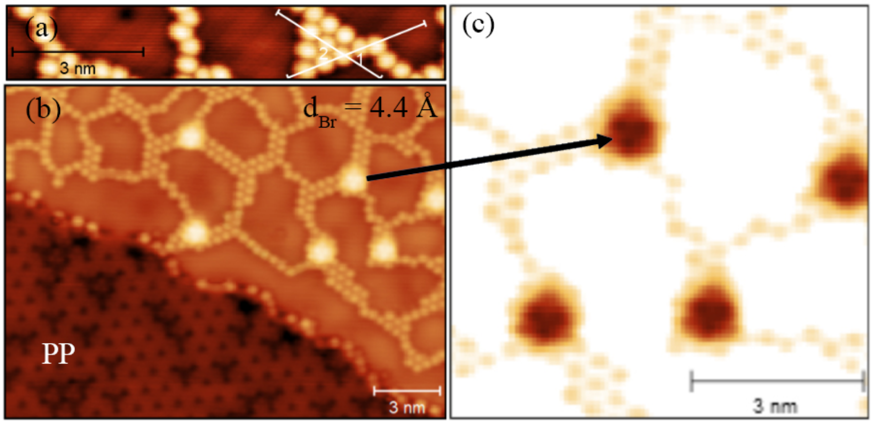

Figure S11: A Br mesh is formed in minor amounts during $CoBr_2$ growth. A closer examination shows that the web is composed of individual circular adsorbates arranged either linearly or in a zigzag pattern. Note the strong similarity to the Br mesh found by Merino-Dìez et. al.[21] We also added a literature reference where a dedicated study was performed for Cl meshes on Au(111) highlighting several similarities.[22]

Triangular Defects of the PP in AFM vs. STM

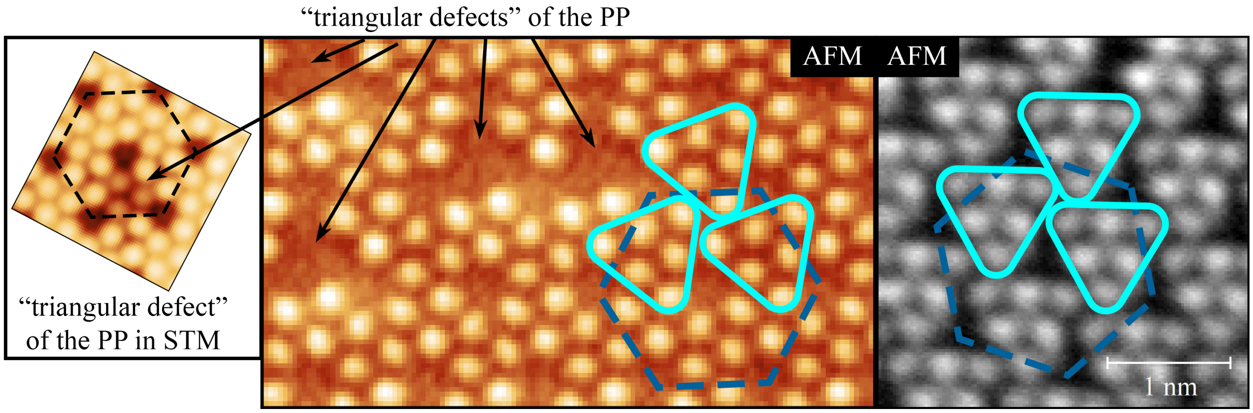

Figure S12: AFM measurements of $CoBr_2$ with two different tip-sample distances (= force acting on the AFM tip). The measurement on the right was obtained with a smaller tip-sample distance. The "triangular defects" marked by the black arrows appear different in AFM than in STM; the three deeper lying atoms are normally not resolved in constant height AFM (only when tip-sample distance is very small).

T-dependence of the Triangular Defects in the PP

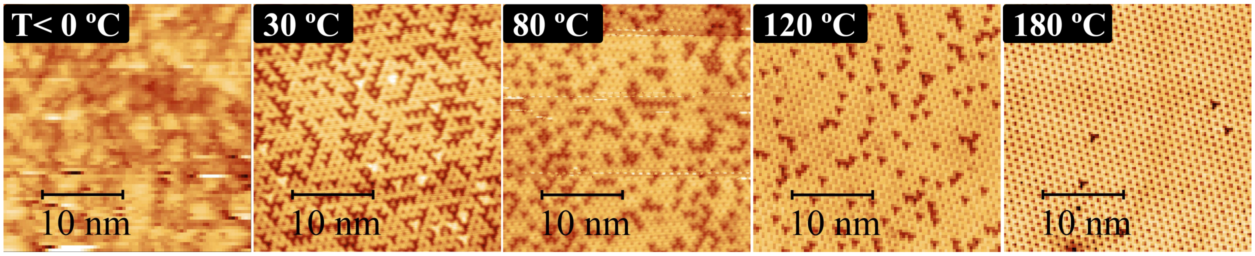

Figure S13: Growth of $CoBr_2$ at different temperatures. Note how the number of triangular defects decreases with increasing deposition temperature. The uncertainty of the temperature is ±20 ° C.

References

(1) Dodecon 4x OMBE Source. 2018; `https://dodecon.de/4xOMBE.html`.

(2) Aldrich, S. Cobalt(II) bromide. 2024; `https://www.sigmaaldrich.com/ES/es/product/aldrich/427136`.

(3) Hadjadj, S. E. et al. Epitaxial Monolayers of the Magnetic 2D Semiconductor FeBr $_2$ Grown on Au(111). Chemistry of Materials **2023**, 35, 9847–9856.

(4) Torbrügge, S.; Schaff, O.; Rychen, J. Application of the KolibriSensor to combined atomic-resolution scanning tunneling microscopy and noncontact atomic-force microscopy imaging. Journal of Vacuum Science & Technology B, Nanotechnology and Microelectronics: Materials, Processing, Measurement, and Phenomena **2010**, 28, C4E12–C4E20.

(5) Larsen, A. H.; Mortensen, J. J.; Blomqvist, J.; Castelli, I. E.; Christensen, R.; Dułak, M.; Friis, J.; Groves, M. N.; Hammer, B.; Hargus, C.; others The atomic simulation environment—a Python library for working with atoms. Journal of Physics: Condensed Matter **2017**, 29, 273002.

(6) Batatia, I.; Benner, P.; Chiang, Y.; Elena, A. M.; Kovács, D. P.; Riebesell, J.; Advincula, X. R.; Asta, M.; Avaylon, M.; Baldwin, W. J.; others A foundation model for atomistic materials chemistry. arXiv preprint arXiv:2401.00096 **2023**,

(7) Grimme, S.; Antony, J.; Ehrlich, S.; Krieg, H. A consistent and accurate ab initio parametrization of density functional dispersion correction (DFT-D) for the 94 elements H-Pu. The Journal of Chemical Physics **2010**, 132.

(8) Jain, A.; Ong, S. P.; Hautier, G.; Chen, W.; Richards, W. D.; Dacek, S.; Cholia, S.; Gunter, D.; Skinner, D.; Ceder, G.; Kristin A., P.; others Commentary: The Materi-

als Project: A materials genome approach to accelerating materials innovation. APL materials **2013**, 1.

(9) Kresse, G.; Hafner, J. Ab initio molecular dynamics for liquid metals. Physical Review B **1993**, 47, 558–561.

(10) Kresse, G.; Furthmüller, J. Efficient iterative schemes for ab initio total-energy calculations using a plane-wave basis set. Physical Review B **1996**, 54, 11169–11186.

(11) Perdew, J. P.; Burke, K.; Ernzerhof, M. Generalized Gradient Approximation Made Simple. Physical Review Letters **1996**, 77, 3865–3868.

(12) Kresse, G.; Joubert, D. From ultrasoft pseudopotentials to the projector augmented-wave method. Physical Review B **1999**, 59, 1758–1775.

(13) Tkatchenko, A.; Scheffler, M. Accurate Molecular Van Der Waals Interactions from Ground-State Electron Density and Free-Atom Reference Data. Physical Review Letters **2009**, 102.

(14) Oinonen, N.; Yakutovich, A. V.; Gallardo, A.; Ondráček, M.; Hapala, P.; Krejčí, O. Advancing scanning probe microscopy simulations: A decade of development in probe-particle models. Computer Physics Communications **2024**, 305, 109341.

(15) Schuler, B.; Liu, W.; Tkatchenko, A.; Moll, N.; Meyer, G.; Mistry, A.; Fox, D.; Gross, L. Adsorption Geometry Determination of Single Molecules by Atomic Force Microscopy. Physical Review Letters **2013**, 111.

(16) Blum, V.; Gehrke, R.; Hanke, F.; Havu, P.; Havu, V.; Ren, X.; Reuter, K.; Scheffler, M. Ab initio molecular simulations with numeric atom-centered orbitals. Computer Physics Communications **2009**, 180, 2175–2196.

(17) Havu, V.; Blum, V.; Havu, P.; Scheffler, M. Efficient integration for all-electron electronic structure calculation using numeric basis functions. Journal of Computational Physics **2009**, 228, 8367–8379.

(18) Botana, A. S.; Norman, M. R. Electronic structure and magnetism of transition metal dihalides: Bulk to monolayer. Physical Review Materials **2019**, 3, 044001.

(19) Thermo Fischer. 2025-02-13; `https://www.thermofisher.com/es/es/home/materials-science/learning-center/periodic-table/transition-metal/cobalt.html`.

(20) Bertel, E.; Netzer, F. Adsorption of bromine on the reconstructed Au(100) surface: LEED, thermal desorption and work function measurements. Surface Science **1980**, 97, 409–424.

(21) Merino-Díez, N.; Pérez Paz, A.; Li, J.; Vilas-Varela, M.; Lawrence, J.; Mohammed, M. S. G.; Berdonces-Layunta, A.; Barragán, A.; Pascual, J. I.; Lobo-Checa, J.; Peña, D.; de Oteyza, D. G. Hierarchy in the Halogen Activation During Surface-Promoted Ullmann Coupling. ChemPhysChem **2019**, 20, 2305–2310.

(22) Cherkez, V. V.; Zheltov, V. V.; Didiot, C.; Kierren, B.; Fagot-Revurat, Y.; Malterre, D.; Andryushechkin, B. V.; Zhidomirov, G. M.; Eltsov, K. N. Self-ordered nanoporous lattice formed by chlorine atoms on Au(111). Physical Review B **2016**, 93.
